# Effect of temperature on antioxidant defense and innate immunity in Brandt’s voles

**DOI:** 10.24272/j.issn.2095-8137.2019.045

**Published:** 2019-07-18

**Authors:** De-Li Xu, Meng-Meng Xu, De-Hua Wang

**Affiliations:** 1 College of Life Sciences, Qufu Normal University, Qufu Shandong 273165, China; 2 State Key Laboratory of Integrated Management of Pest Insects and Rodents, Institute of Zoology, Chinese Academy of Sciences, Beijing 100101, China

**Keywords:** Antioxidant defense, Brandt’s voles (*Lasiopodomys brandtii*), Immunity, Temperature

## Abstract

Ambient temperature is an important factor influencing many physiological processes, including antioxidant defense and immunity. In the present study, we tested the hypothesis that antioxidant defense and immunity are suppressed by high and low temperature treatment in Brandt’s voles (*Lasiopodomys brandtii*). Thirty male voles were randomly assigned into different temperature groups (4, 23, and 32 °C, *n*=10 for each group), with the treatment course lasting for 27 d. Results showed that low temperature increased gross energy intake (GEI) and liver, heart, and kidney mass, but decreased body fat mass and dry carcass mass. With the decline in temperature, hydrogen peroxide (H_2_O_2_) concentration, which is indicative of reactive oxygen species (ROS) levels, increased in the liver, decreased in the heart, and was unchanged in the kidney, testis, and small intestine. Lipid peroxidation indicated by malonaldehyde (MDA) content in the liver, heart, kidney, testis, and small intestine did not differ among groups, implying that high and low temperature did not cause oxidative damage. Similarly, superoxide dismutase (SOD) and catalase (CAT) activities and total antioxidant capacity (T-AOC) in the five tissues did not respond to low or high temperature, except for elevation of CAT activity in the testis upon cold exposure. Bacteria killing capacity, which is indicative of innate immunity, was nearly suppressed in the 4 °C group in contrast to the 23 °C group, whereas spleen mass and white blood cells were unaffected by temperature treatment. The levels of testosterone, but not corticosterone, were influenced by temperature treatment, though neither were correlated with innate immunity, H_2_O_2_ and MDA levels, or SOD, CAT, and T-AOC activity in any detected tissues. Overall, these results showed that temperature had different influences on oxidative stress, antioxidant enzymes, and immunity, which depended on the tissues and parameters tested. Up-regulation or maintenance of antioxidant defense might be an important mechanism for voles to survive highly variable environmental temperatures.

## INTRODUCTION

Ambient temperature is an important factor influencing many physiological processes in animals, including antioxidant defense and immune function (King, 2004; Marnila & Lilius, 2015). While antioxidant defense can eliminate reactive oxygen species (ROS), oxidative stress can occur when ROS production overwhelms antioxidant capacity (Dickinson & Chang, 2011; Selman et al., 2013). Oxidative stress can cause oxidative damage to biomolecules (i.e., lipids, proteins, and DNA) and is deleterious to the structure and function of cells and tissues (Marri & Richner, 2015; Raut et al., 2012). The immune system, which protects animals from infection and pathogens, plays a key role in survival and fitness (Owens & Wilson, 1999; Sheldon &Verhulst, 1996). Therefore, both antioxidant capacity and immune function are crucial for animals to maintain health and survival; however, both are markedly influenced by environmental temperature (Carroll et al., 2012; Metcalfe & Alonso-Alvarez, 2010; Zhou et al., 2015).

Several researchers have investigated the impact of temperature on antioxidant defense. For instance, cold exposure in rats has been shown to increase lipid peroxidation in the brain but decrease it in the liver (Lomakina, 1980) and enhance superoxide dismutase (SOD) activity in the heart and kidney but suppress it in the lungs and pancreas (Vasilijević et al., 2007; Yuksel et al., 2008). Moreover, cold stress is reported to increase protein oxidation in the liver and muscle but not affect brown adipose tissue in short-tailed field voles (*Microtus agrestis*) (Selman et al., 2000, 2002, 2008). Lipid peroxidation, total antioxidant capacity (T-AOC), and glutathione peroxidase activity in brown adipose tissue has been shown to increase under low temperature but decrease under high temperature in striped hamsters (*Cricetulus barabensis*) (Zhou et al., 2015). Furthermore, high temperature exposure has been shown to cause oxidative damage in broiler chickens (Tan et al., 2010) and decrease SOD activity in rat testes (Kanter et al., 2013).

Bacteria killing capacity, which entails phagocytes, opsonizing proteins, and natural antibodies acting against specific pathogens, has been used to evaluate innate immunity in mammals (Demas et al., 2011; Tieleman et al., 2005). Immune organs and total white blood cells (WBC) are indirect parameters indicative of immune function (Calder & Kew, 2002). A larger spleen is representative of a stronger immune system (Smith & Hunt, 2004), and adipose tissue is no longer regarded as a simple passive energy reserve but also as an important endocrine and immune organ (Ahima & Flier, 2000; Fantuzzi, 2005; Trayhurn, 2005). Although investigators have examined the impact of temperature on immunity in laboratory animals, including mice (Xu et al., 1992) and rats (Kozyreva & Eliseeva, 2000, 2004), research on wild rodents remains scarce or contradictory. For instance, both cellular immunity and bacteria killing capacity are reported to be unaffected by low and high temperature in female Mongolian gerbils (*Meriones unguiculatus*) (Yang et al., 2013).

Stressful conditions, such as cold or hot temperatures, can stimulate the hypothalamic-pituitary-adrenal axis, and hence secretion of glucocorticoids such as corticosterone, which is related to oxidative damage and immunity (Kim et al., 2013; Sapolsky et al., 2000). Moreover, testosterone incurs oxidative costs such as increased production of ROS according to the oxidation handicap hypothesis (Alonso-Alvarez et al., 2007, 2008). Testosterone also has suppressive effects on immune function in many species, including mammals and birds (Trigunaite et al., 2015).

Brandt’s voles (*Lasiopodomys brandtii*) are primarily distributed in the grasslands of Inner Mongolia in China as well as the Republic of Mongolia and the Baikal region of Russia (Li et al., 2010; Walker, 1968). They are strictly herbivorous and feed mainly on grass (Wang et al., 2003). The climate is arid and characterized by warm, dry summers (maximum 42.6 °C) and cold winters (minimum -47.5 °C) (Chen, 1988; Zhao & Wang, 2006). Thus, this species experiences considerable seasonal fluctuations in temperature, photoperiod, and food availability (Wang et al., 2000; Zhang & Wang, 1998). Previous research has shown that resting metabolic rate (RMR) and uncoupling protein 1 (UCP1) content increase but body fat mass decreases in voles upon cold exposure (Liu et al., 2009; Wang, 2007). Both immune responses (Demas, 2004; Martin et al., 2003) and oxidative stress come at a cost (Dowling & Simmons, 2009) and life-history trade-off (Hall et al., 2010; Martin et al., 2007; Monaghan et al., 2009). Therefore, we expect that low and high temperature exposure should cause oxidative stress and immunosuppression in voles.

## MATERIAL AND METHODS

### Animals and experimental design

Animals used in this study were the offspring of a captive colony trapped on the Inner Mongolian grasslands in May 1999 and brought to the animal facility at the Institute of Zoology, Chinese Academy of Sciences, Beijing, China. All animal procedures were licensed under the Institutional Animal Care and Use Committee of the Institute of Zoology, Chinese Academy of Sciences (approval number: QFNUDW2012016; approval date: 20120628). The animals were housed individually after weaning in plastic cages (30 cm×15 cm×20 cm) with sawdust as bedding under a constant photoperiod (16 h:8 h light-dark cycle) and temperature (23±1 °C). Rabbit pellet chow and water were provided *ad libitum*. Thirty male voles (aged 4–6 months) were randomly assigned into the cold (4±1 °C) (*n*=10), warm (23±1 °C) (*n*=10), and hot groups (32±1 °C) (*n*=10) (hereafter referred to as the 4 °C, 23 °C, and 32 °C groups). The treatment course lasted for 27 d. One vole escaped after 13 d of cold treatment and another vole died after 16 d of cold treatment. Therefore, these two voles were not included in subsequent statistical analyses.

### Energy intake

Body mass was recorded every 3 d and energy budgets were determined at 3-d intervals over the course of the study. Food intake was measured in metabolic cages, as described previously (Xu & Wang, 2011; Xu et al., 2011). Food was provided quantitatively. Food residue and feces were collected from each subject over 3 d before acclimation began and during the course of treatment and were separated after they were dried at 60 °C to a constant mass (Liu et al., 2003). Energy content of the food and feces was determined using a Parr 1281 oxygen bomb calorimeter (Parr Instrument, USA). Gross energy intake (GEI) was calculated according to Grodzinski & Wunder (1975) and Liu et al. (2003) using the following equation:

### GEI (kJ/d)=dry matter intake (DMI)×

energy content of food (kJ/g) (1)

### Organ and body composition

At the end of the experiment, animals were sacrificed by CO_2_ asphyxiation, after which trunk blood was collected for later measurement of WBC. Blood samples were allowed to clot for 1 h and centrifuged at 4 °C for 30 min at 4 000 r/min. Sera were collected and stored in polypropylene microcentrifuge tubes at -80 °C for later hormone (i.e., corticosterone and testosterone) and bacterial killing capacity assays. Organ mass was measured as described previously (Xu & Wang, 2011; Xu et al., 2011). In brief, visceral organs, including the heart, liver, kidneys, testes, small intestine, and spleen, were dissected on an ice box and weighed (±1 mg). The length of the small intestine was measured by extending the organ to its unstressed length along a ruler (±1 mm) (Pei et al., 2001). The small intestine was then opened and rinsed with saline to eliminate all gut contents, blotted dry on tissue paper, and weighed. The heart, liver, kidneys, testes, and small intestine were stored at -80 °C for later antioxidant enzyme assays. The carcass was dried in an oven at 60 °C to a constant mass, and then weighed again to obtain dry mass. The difference between the wet and dry carcass mass was the water mass of the carcass. Total body fat was extracted from the dried carcass by petroleum ether extraction in a Soxhlet apparatus, and body fat content was calculated as the proportion of total body fat mass divided by wet carcass mass (Xu & Wang, 2010).

### Oxidative stress marker assays

Lipid peroxidation, which is indicative of oxidative damage, was examined as described previously (Yang et al., 2013). Specifically, lipid peroxidation was evaluated by quantifying malonaldehyde (MDA) (Del Rio et al., 2005) using a thiobarbituric acid reactive substances (TBARS) assay kit (Nanjing Jiancheng, Nanjing, China) following the manufacturer’s instructions. The absorbance of the eluent was monitored spectrophotometrically at 532 nm (BioTek Synergy 4 Hybrid Microplate Reader, BioTek, Winooski, Vermont, USA). The intra- and inter-assay coefficients of variation for this assay were <1.5% and <3.32%, respectively. Lipid peroxidation was expressed as nmol MDA per mg protein.

ROS levels were measured in the tissues by examining hydrogen peroxide (H_2_O_2_) levels (Zhou et al., 2015). The H_2_O_2_ levels were analyzed using a commercial kit (Nanjing Jiancheng, Nanjing, China) in accordance with the manufacturer’s instructions. Levels of H_2_O_2_ were expressed as μmol/g protein.

### Antioxidant enzymes

The activities of antioxidant enzymes, including SOD and CAT, and total antioxidant capacity (T-AOC) were also determined using commercial kits (Nanjing Jiancheng, Nanjing, China) according to the manufacturer’s instructions. One unit of SOD was defined as the amount of enzyme that caused 50% inhibition of superoxide radical produced by the reaction between xanthine and xanthine oxidase at 37 °C; one unit of CAT activity was defined as the decomposition of 1 μmol H_2_O_2_ per min; one unit of T-AOC was defined as the extent to which optical density increased by 0.01 per milligram protein per min (Chen et al., 2014).

### Immunological parameters

WBC count was determined as described previously (Xu & Wang, 2010). In brief, 20 μL of whole blood was diluted immediately in a 0.38 mL solution containing 1.5% glacial acetic acid and 1% crystal violet (Sigma). The leukocytes were then counted in an improved Neubauer chamber using a microscope. Total number of WBC was determined by counting all leucocytes in the four large corner squares of the Neubauer chamber and multiplying the raw data by 5×10^7^ to obtain final values (10^9^ cells/L) (Yang, 2004).

Serum bacterial killing capacity, which is indicative of innate immunity, was performed in a sterile laminar flow cabinet to assess the functional response by the animal’s innate immune system against a relevant pathogen, *Escherichia coli* (Demas et al., 2011; Tieleman et al., 2005; Yang et al., 2013). Briefly, serum samples were diluted 1: 20 in a CO_2_-independent medium (Gibco no. 18045, Carlsbad, GA, USA). A standard number of colony-forming units (CFUs) of *E. coli* (ATCC no. 8739, Microbial Culture Collection Center of Guangdong Institute of Microbiology, China) was added to each sample at a ratio of 1:10, after which the mixture was allowed to incubate at 37 °C for 30 min to induce bacterial killing. After incubation, 50 μL of each sample was added to tryptic soy agar plates in duplicate. All plates were covered and left to incubate upside down at 37 °C for 24 h. After this, total CFUs were counted and bactericidal capacity was calculated as 100% minus the mean number of CFUs for each sample divided by the mean number of CFUs for the positive controls (containing only medium and standard bacterial solution), i.e., the percentage of bacteria killed relative to the positive control.

### Serum corticosterone assays

Serum corticosterone concentrations were determined using a rat corticosterone ELISA kit (Cat. No. HR083, RapidBio Lab. Calabasas, California, USA). The lowest level of corticosterone that could be detected by this assay was 1.0 nmol/L. All procedures were in accordance with the manufacturer’s instructions. Inter- and intra-assay variabilities for corticosterone were <1.1% and 7.5%, respectively.

### Serum testosterone

Serum testosterone concentrations were assessed using a rat testosterone ELISA kit (Cat. No. HR083, RapidBio Lab. Calabasas, California, USA) following the manufacturer’s instructions. The tested range of testosterone was 0.13–25.6 ng/mL. Intra- and inter-assay variabilities for testosterone were <9.0% and 11.0%, respectively.

### Statistical analysis

Data were analyzed using SPSS 13.0 software (SPSS Inc., Chicago, IL, USA). Prior to all statistical analyses, data were examined for normality and homogeneity of variance using Kolmogorov-Smirnov and Levene tests, respectively. Changes in body mass and GEI during the experiment were analyzed with the Repeated Measure of General Linear Model (GLM). Differences in body mass at any time point, MDA and H_2_O_2_ levels, SOD and CAT activity, T-AOC, WBC, innate immunity, and corticosterone and testosterone concentrations in different groups were analyzed by one-way analysis of variance (ANOVA) followed by Tukey’s *post hoc* tests. Group differences in wet organ mass and GEI, with body mass as the covariate, at any time point were analyzed by a one-way analysis of covariance (ANCOVA) followed by Bonferroni *post-hoc* tests. Significant group differences were further evaluated by GLM multivariate analysis followed by Bonferroni *post hoc* tests. Pearson correlation analysis was performed to determine the correlations of corticosterone, testosterone, and antioxidant parameters. Results are expressed as means±*SE*, with *P*<0.05 considered to be statistically significant.

## RESULTS

### Body mass and energy intake

**Figure 1:**
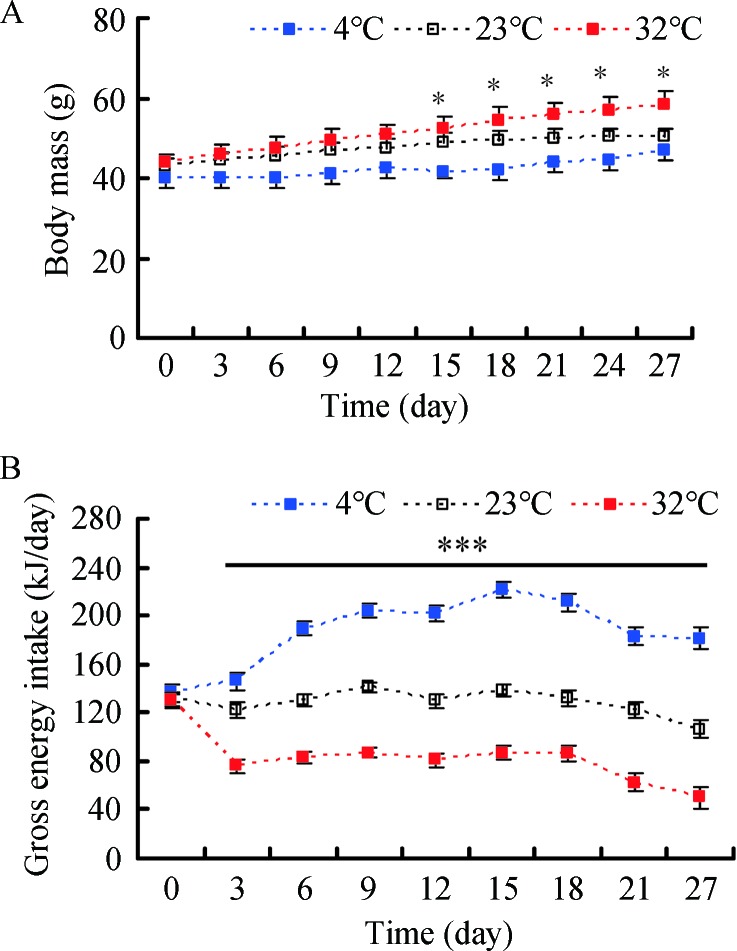
**Effect of temperature on body mass (A) and gross energy intake (B) in Brandt**’**s voles acclimated to 4 °C, 23 °C, and 32 °C** Data are means±*SE*. *: *P<*0.05, ***: *P<*0.001.

Body mass among the 4 °C, 23 °C, and 32 °C groups did not differ significantly before the experiment began (day 0, *F*_2, 25_=0.650, *P*=0.531) (Figure 1A). Based on repeated measure ANOVA, body mass changed significantly over the 27 d acclimation to low and high temperature (*F*_9, 225_=40.915, *P*<0.001). Body mass among the three groups show no significant differences from day 3 (*F*_2, 25_=1.477, *P*=0.248) to day 12 (*F*_2, 25_=2.822, *P*=0.078), whereas body mass differed significantly from day 15 (*F*_2, 25_=4.600, *P*=0.020) to day 27 (*F*_2, 25_=4.203, *P*=0.027) (Figure 1A). At the end of the experiment, body mass in the 4 °C, 23 °C, and 32 °C groups increased by 16.2%, 17.4%, and 32.4%, respectively, compared with body mass on day 0 (Table 1). There was no difference in GEI among the three groups on day 0 (*F*_2, 24_=0.251, *P*=0.780) (Figure 1B); however, GEI changed significantly over the course of the experiment (*F*_8, 200_=54.430, *P<*0.001). GEI was significantly influenced by temperature from day 3 to day 27 and was significantly higher in the 4 °C group and lower in the 32 °C group relative to the 23 °C group (day 3, *F*_2, 24_=10657.8, *P<*0.001; day 27, *F*_2, 24_=22.7, *P<*0.001) (Figure 1B).

**Table 1:** **Effect of temperature on body composition and wet organ mass in Brandt**’**s voles**

**Parameter**	**4 °C**	**23 °C**	**32 °C**	**Statistical summary**	
				***F*_2, 25_**	***P***
**Body composition** (g)					
Initial body mass	40.2±2.7	43.0±1.8	43.8±2.3	0.650	0.531
Final body mass	46.7±2.1^b^	50.5±6.6^ab^	58.0±3.6^a^	4.203	0.027
Wet carcass	30.3±2.1^b^	35.0±1.6^b^	42.4±2.6^a^	7.819	0.002
Dry carcass	13.2±2.8^b^	16.5±1.3^ab^	22.3±2.3^a^	6.995	0.004
Body water	17.1±1.2	18.5±0.7	20.1±0.8	2.690	0.087
Fat free dry carcass	9.1±0.5^b^	9.9±0.4^ab^	11.5±0.6^a^	6.090	0.007
Body fat mass	4.1±0.5^b^	6.6±1.1^ab^	10.7±1.8^a^	6.255	0.006
Fat content (%)	30.3±1.6^b^	38.1±3.1^ab^	45.3±3.2^a^	6.584	0.005
**Organ mass** (mg)				***F*_2, 24_**	***P***
Heart	249±8^a^	213±6^b^	178±7^c^	18.116	<0.001
Liver	2331±136	1905±109	1869±125	3.617	0.042
Kidney	618±24^a^	510±19^b^	399±22^c^	18.282	<0.001
Small intestine	312±79	344±63	259±73	0.369	0.695
Small intestine length (cm)	35.1±1.0^a^	32.9±0.8^a^	29.4±0.9 ^b^	7.983	0.002
Testis	813±74	929±59	796±68	1.437	0.257
Spleen	35±3	32±3	36±3	0.748	0.484

Values are means±*SE*. Values for a specific parameter that share different superscripts are significantly different at *P*<0.05. Body composition was analyzed by one-way ANOVA, and organ mass was determined by General Linear Model multivariate analysis followed by Bonferroni *post hoc* tests with body mass as the covariate.

### Body composition and organs

Body composition (i.e., wet carcass, dry carcass, fat free dry carcass, body fat mass, and fat content) decreased, whereas organ mass (i.e., heart, liver, and kidney) and small intestine length increased, and spleen, testis, and small intestine mass remained unchanged with the decline in temperature (Table 1). Body fat mass was 37.9% lower in the 4 °C group and 62.1% higher in the 32 °C group in contrast with the 23 °C group.

### Oxidative stress

**Figure 2:**
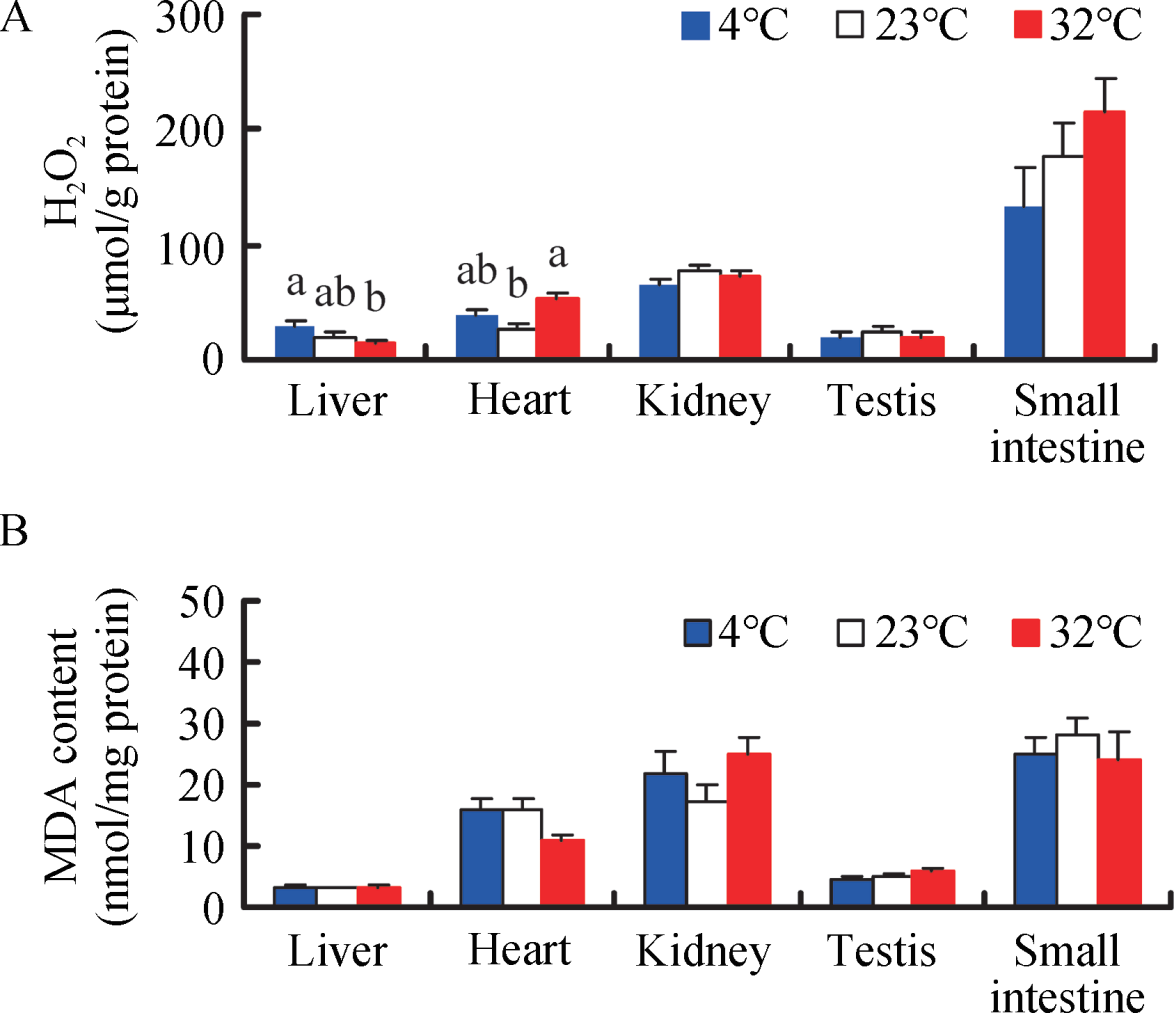
**Effect of temperature on H_2_O_2_ (A) and MDA levels (B) in Brandt**’**s voles acclimated to 4 °C, 23 °C, and 32 °C** Different letters above columns indicate significant differences at *P<*0.05.

H_2_O_2_ levels showed significant differences among the various tissues in all three treatment groups (*F*_4, 135_=58.61, *P<*0.001, Figure 2A). In the 4 °C group, H_2_O_2_ levels were significantly higher in the small intestine than in the liver, heart, kidney, or testis (*post hoc*, *P<*0.05). Temperature had a significant effect on H_2_O_2_ levels in the liver, which were 46.7% higher in the 4 °C group and 27.9% lower in the 32 °C group compared to the 23 °C group (*F*_2, 25_=4.584, *P*=0.020). H_2_O_2_ levels in the heart were also affected significantly by temperature and were 44.3% and 102.9% higher in the 4 °C and 32 °C groups, respectively, compared to the 23 °C group (*F*_2, 25_=7.949, *P*=0.002). However, temperature had no effect on H_2_O_2_ levels in the kidney (*F*_2, 25_=1.506, *P*=0.241), testis (*F*_2, 25_=0.166, *P*=0.848), or small intestine (*F*_2, 25_=1.649, *P*=0.212) (Figure 2A).

MDA levels differed significantly among the various tissues (*F*_4, 135_=59.29, *P<*0.001, Figure 2B), and were significantly higher in the kidney and small intestine than that in the liver, heart, or testis in the 4 °C group (*post hoc*, *P<*0.05). Temperature had no influence on MDA content in the liver (*F*_2, 25_=0.247, *P*=0.783), kidney (*F*_2, 25_=1.816, *P*=0.184), testis (*F*_2, 25_=2.039, *P*=0.151), or small intestine (*F*_2, 25_=0.356, *P*=0.704). However, MDA levels in the heart were 31.5% lower in the 32 °C group than that in the 23 °C group (*F*_2, 25_=3.391, *P*=0.050) (Figure 2B).

### Antioxidant enzymes

**Figure 3:**
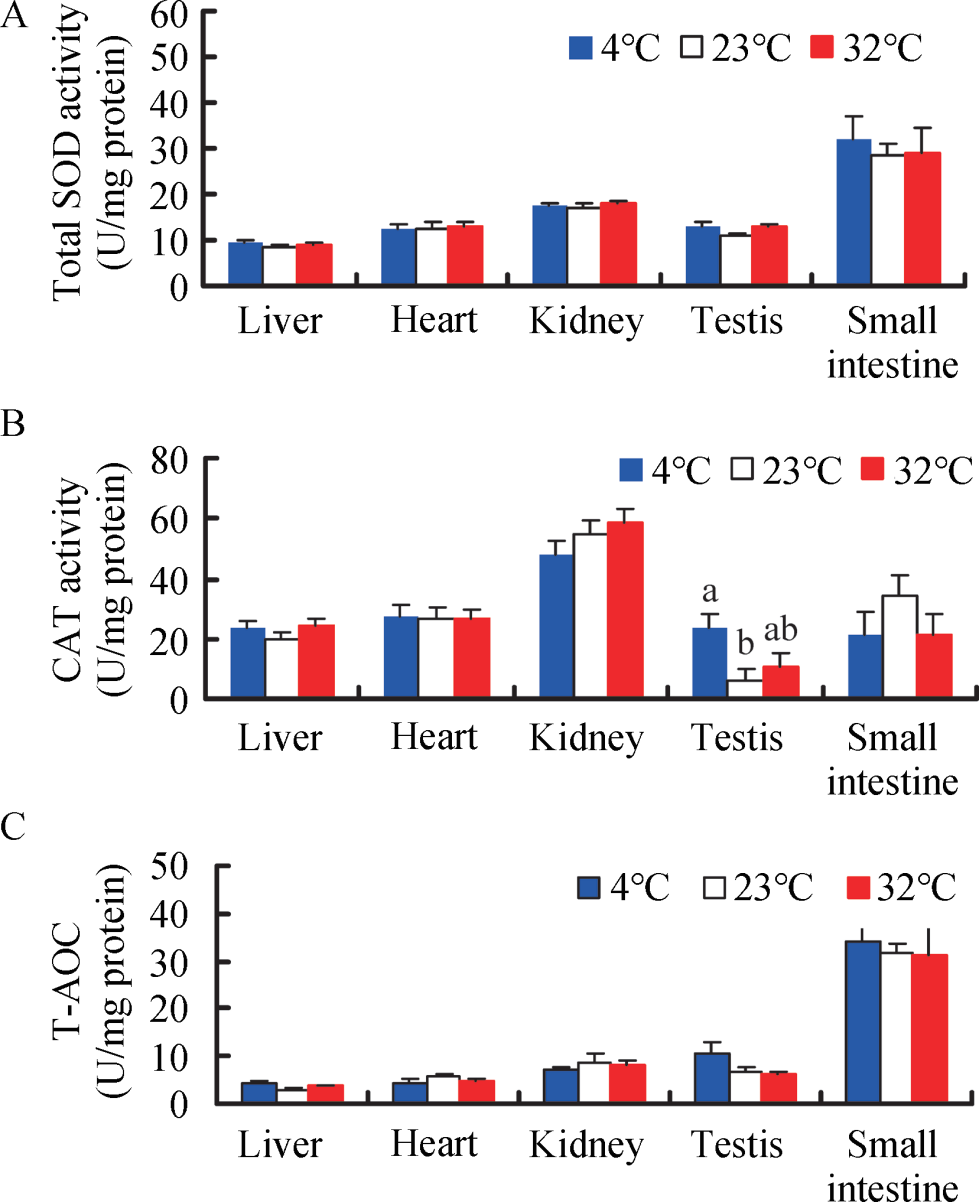
**Effect of temperature on total SOD activity (A), CAT activity (B), and total antioxidant capacity (T-AOC) (C) in Brandt**’**s voles** Different letters above columns indicate significant differences at *P<*0.05.

A significant difference in total SOD activity was found among the five tissues (*F*_4, 135_=42.64, *P<*0.001, Figure 3A), with higher activity detected in the small intestine compared with the other four tissues (*post hoc*, *P<*0.05). There were no significant differences in total SOD activity in the liver (*F*_2, 25_=0.531, *P*=0.594), heart (*F*_2, 25_=0.076, *P*=0.927), kidney (*F*_2, 25_=0.523, *P*=0.599), and small intestine (*F*_2, 25_=0.184, *P*=0.833) among the 4 °C, 23 °C, and 32°C groups. Total SOD activity in the testis was 18.8% and 19.9% higher in the 4 °C and 32 °C groups, respectively, compared to the 23 °C group (*F*_2, 25_=3.239, *P*=0.056) (Figure 3A).

CAT activity exhibited significant differences among the five tissues (*F*_4, 135_=31.17, *P<*0.001, Figure 3B), and was higher in the kidney than the other four tissues in the 4 °C group (*post hoc*, *P<*0.05). CAT activity in the testis was significantly increased in the 4 °C (304.6%) and 32 °C groups (87.3%), respectively, compared to the 23 °C group (Figure 3B). However, temperature had no significant impact on CAT activity in the liver, heart, kidney, or small intestine (liver, *F*_2, 25_=1.165, *P*=0.328; heart, *F*_2, 25_=0.016, *P*=0.984; kidney, *F*_2, 25_=1.299, *P*=0.291; small intestine, *F*_2, 25_=1.110, *P*=0.345) (Figure 3B).

T-AOC differed significantly among the five tissues (*F*_4, 135_=59.44, *P<*0.001, Figure 3C), and was higher in the small intestine than that in the other four tissues in the 4 °C group (*post hoc*, *P<*0.05). No significant differences in T-AOC were observed in the liver (*F*_2, 25_=2.313, *P*=0.120), heart (*F*_2, 25_=0.683, *P*=0.514), kidney (*F*_2, 25_=0.437, *P*=0.651), testis (*F*_2, 25_=2.368, *P*=0.114), and small intestine (*F*_2, 25_=0.068, *P*=0.934) among the groups (Figure 3C). However, significant positive correlations were found between MDA levels and SOD, CAT, and T-AOC activities in the heart and small intestine and between H_2_O_2_ levels and MDA, SOD, and T-AOC activities in the small intestine, with some significant correlations found in the liver, kidney, and testis (Table 2).

**Table 2:** **Pearson**’**s correlation coefficients between H_2_O_2_ and MDA levels, SOD, CAT, T-AOC activity, and corticosterone and testosterone in Brandt**’**s voles**

		H_2_O_2_	MDA	SOD	CAT	T-AOC	CORT	T
Liver	H_2_O_2_	1						
	MDA	–0.096	1					
	SOD	–0.042	0.435*	1				
	CAT	0.025	0.343	0.592**	1			
	T-AOC	0.108	0.179	0.442*	0.402*	1		
	CORT	–0.352	–0.106	–0.088	–0.044	0.140	1	
	T	0.258	–0.005	0.136	0.102	–0.123	–0.840**	1
		H_2_O_2_	MDA	SOD	CAT	T-AOC	CORT	T
Heart	H_2_O_2_	1						
	MDA	–0.045	1					
	SOD	0.346	0.390*	1				
	CAT	0.107	0.520**	0.551**	1			
	T-AOC	0.087	0.566**	0.695**	0.451*	1		
	CORT	–0.085	0.208	0.056	0.089	0.219	1	
	T	0.273	–0.206	0.048	–0.057	–0.037	–0.840**	1
		H_2_O_2_	MDA	SOD	CAT	T-AOC	CORT	T
Kidney	H_2_O_2_	1						
	MDA	0.041	1					
	SOD	–0.187	–0.116	1				
	CAT	0.173	0.335	0.169	1			
	T-AOC	0.148	0.266	0.355	0.330	1		
	CORT	0.117	–0.183	–0.158	0.121	–0.023	1	
	T	–0.068	0.114	0.209	–0.114	–0.101	–0.840**	1
		H_2_O_2_	MDA	SOD	CAT	T-AOC	CORT	T
Testis	H_2_O_2_	1						
	MDA	0.248	1					
	SOD	0.212	0.324	1				
	CAT	–0.200	–0.231	0.233	1			
	T-AOC	0.228	–0.197	0.536**	0.071	1		
	CORT	–0.163	–0.322	–0.447*	–0.171	–0.207	1	
	T	0.129	0.339	0.392*	0.249	–0.036	–0.840**	1
		H_2_O_2_	MDA	SOD	CAT	T-AOC	CORT	T
SI	H_2_O_2_	1						
	MDA	0.507**	1					
	SOD	0.651**	0.636**	1				
	CAT	0.340	0.411*	0.397*	1			
	T-AOC	0.503**	0.633**	0.794**	0.329	1		
	CORT	–0.008	0.162	–0.005	0.208	–0.194	1	
	方正汇总行方正汇总行T	–0.022	–0.204	–0.111	–0.173	–0.002	–0.840**	1

SI: Small intestine; CORT: Corticosterone; T: Testosterone. *: *P<*0.05, **: *P<*0.01.

### Immunological parameters

**Figure 4:**
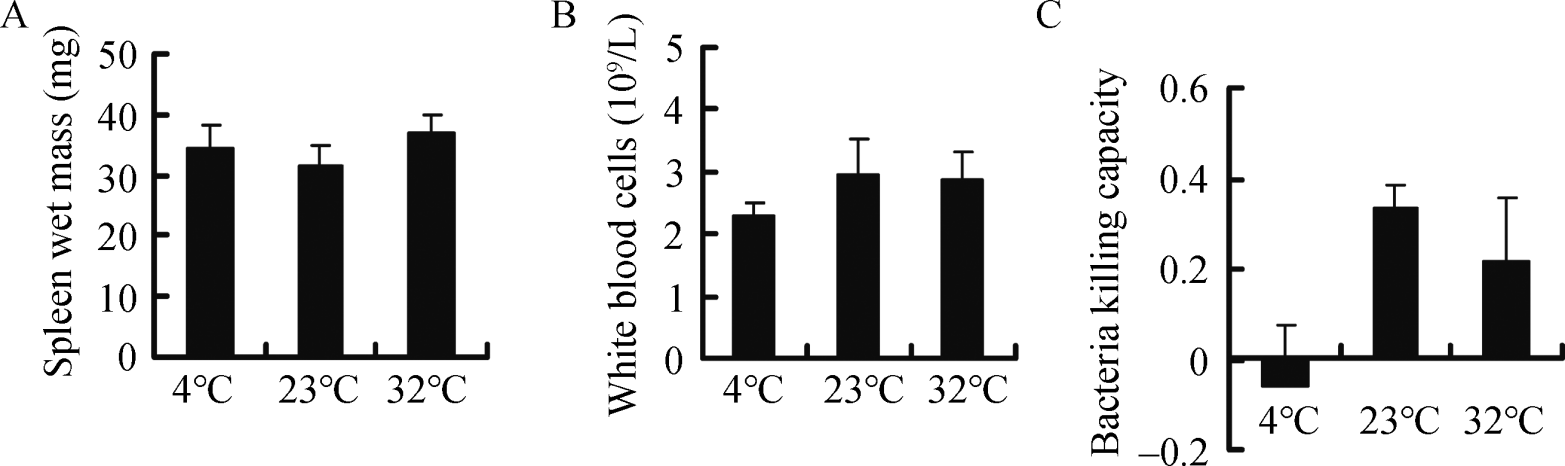
**Effect of temperature on spleen wet mass (A), white blood cell count (B), and bacteria killing capacity (C) in Brandt**’**s voles**

No statistically significant differences were observed in wet spleen mass (*F*_2, 25_=0.748 *P*=0.484) (Figure 4A), WBC (*F*_2, 25_=0.504, *P*=0.610) (Figure 4B), or bacteria killing capacity (*F*_2, 25_=2.822, *P*=0.079) (Figure 4C). Innate immunity was 118.0% and 35.5% lower in the 4 °C and 32 °C groups, respectively, compared to the 23 °C group. Innate immunity was negatively correlated with liver H_2_O_2_ levels and kidney MDA content, but not with body fat mass, H_2_O_2_ levels, or MDA content in other tissues (Table 3).

**Table 3:** **Pearson**’**s correlation coefficients between innate immunity, H_2_O_2_ and MDA levels, corticosterone, testosterone, and body fat mass in Brandt**’**s voles**

		Immunity	H_2_O_2_					MDA							
			Liver	Heart	Kidney	Testis	SI	Liver	Heart	Kidney	Testis	SI	CORT	T	Fat
Immunity		1													
H_2_O_2_	Liver	–0.441*	1												
	Heart	–0.143	–0.030	1											
	Kidney	–0.105	–0.181	–0.349	1										
	Testis	0.050	–0.066	0.229	–0.104	1									
	SI	0.032	–0.259	–0.006	–0.078	–0.061	1								
MDA	Liver	–0.171	–0.096	–0.021	0.023	–0.069	0.261	1							
	Heart	0.011	0.200	–0.045	–0.071	0.172	–0.342	–0.527**	1						
	Kidney	–0.423*	0.333	0.383*	0.041	0.000	0.078	0.099	–0.200	1					
	Testis	–0.032	0.008	0.161	–0.014	0.248	0.269	–0.121	–0.203	–0.039	1				
	SI	0.185	–0.098	–0.408	0.129	–0.114	0.507**	0.186	–0.089	–0.160	–0.075	1			
	CORT	0.206	–0.352	–0.085	0.117	–0.163	–0.008	–0.106	0.208	–0.183	–0.322	0.162	1		
	T	–0.119	0.258	0.273	–0.068	0.129	–0.022	–0.005	–0.206	0.114	0.339	–0.204	–0.840**	1	
	Fat	–0.065	–0.219	0.289	0.163	0.005	0.085	–0.435*	0.089	0.393*	0.253	–0.291	–0.111	0.132	1

SI: Small intestine; CORT: Corticosterone; T: Testosterone; Fat: Body fat mass. *: *P<*0.05, **: *P<*0.01.

### Serum corticosterone

**Figure 5:**
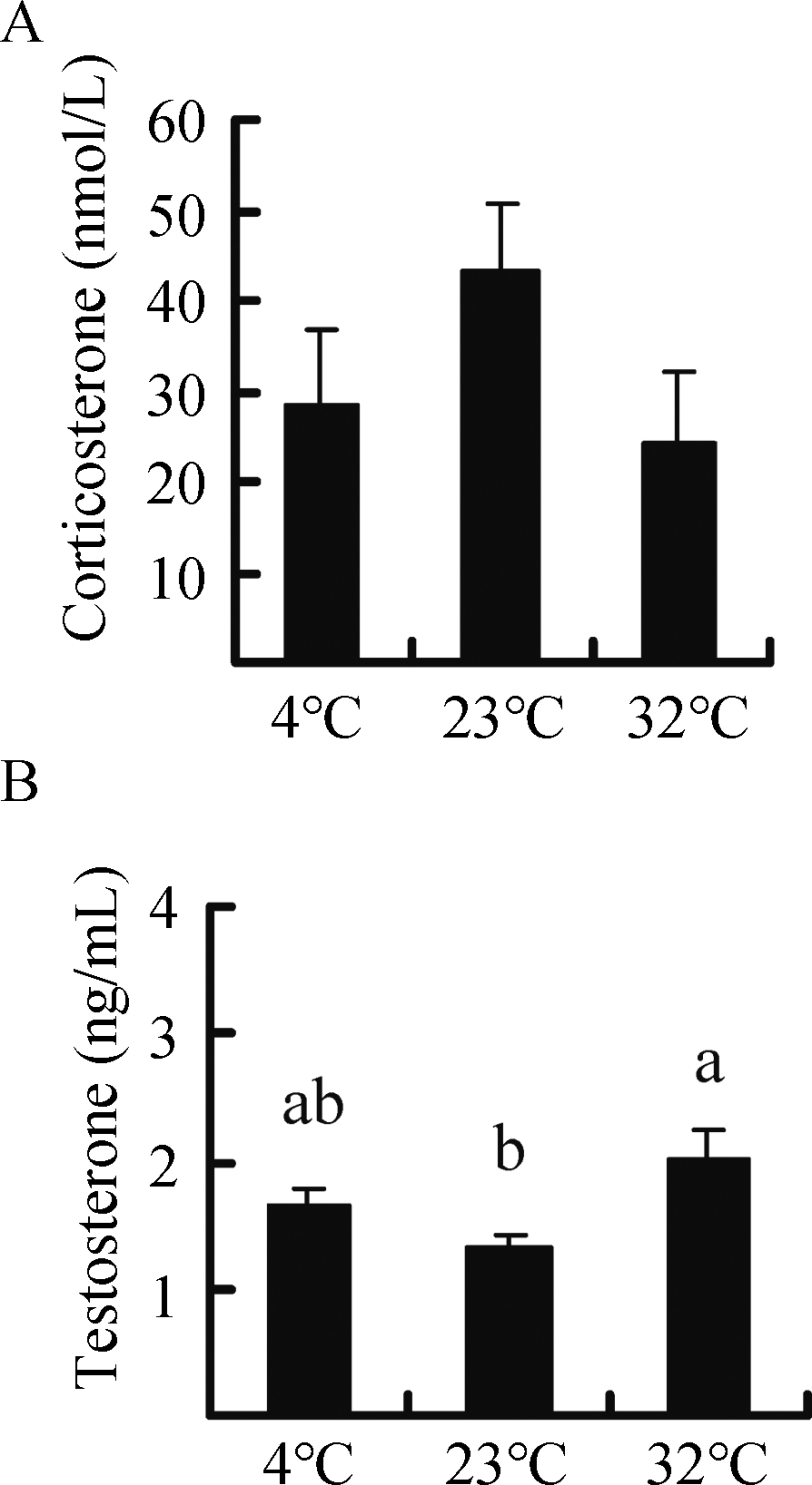
**Effect of temperature on the concentration of corticosterone (A) and testosterone (B) in Brandt**’**s voles acclimated to 4 °C, 23 °C, and 32 °C**

Corticosterone concentration did not differ among the groups (*F*_2, 25_=1.818, *P*=0.183) (Figure 5A). Furthermore, corticosterone concentration was not correlated with H_2_O_2_ levels, MDA content, or SOD, CAT, and T-AOC activities in the liver, heart, kidney, testis, or small intestine, except for a negative correlation between corticosterone and SOD activity in the testis (Table 2). Moreover, no significant correlation was found between innate immunity and corticosterone concentration (*r*=0.206, *P*=0.293) (Table 3).

### Serum testosterone

Testosterone concentration was significantly influenced by temperature and was 25.8% and 53.1% higher in the 4 °C and 32 °C groups, respectively, compared to the 23 °C group (*F*_2, 25_=4.403, *P*=0.023) (Figure 5B). Testosterone concentration was not correlated with H_2_O_2_ levels, MDA content, or SOD, CAT, and T-AOC activities in the liver, heart, kidney, testis, or small intestine, except for a positive correlation with testis SOD (Table 2). Testosterone concentration was negatively correlated with corticosterone concentration (Table 2) but was not correlated with innate immunity (*r*=–0.119, *P*=0.546) (Table 3).

## DISCUSSION

Ambient temperature is one of the most important factors affecting physiological processes. Contrary to our expectation, we found that temperature exerted different influences on oxidative stress, antioxidant enzymes, and immunity depending on the tissues and parameters tested. With the decline in temperature, ROS levels increased in the liver but did not change in the kidney, testis, or small intestine. ROS levels in the heart were higher in the 32 °C group than in the 23 °C group. Compared to the 23 °C group, cold exposure elevated CAT activity in the testis but not in the liver, heart, kidney, or small intestine. MDA content and SOD and T-AOC activities in the five tissues showed no response to low or high temperature. Bacteria killing capacity was nearly suppressed in the 4 °C group compared with that in the 23°C group, whereas spleen mass and WBC were unaffected by temperature treatment.

### Body mass, body composition, and organs

To satisfy the augmentation in energy requirements during cold temperature, animals usually increase energy intake (Hammond & Wunder, 1995). In the present study, we also found that gross energy intake increased in the voles with the decline in temperature. However, the body masses in the 4 °C and 32 °C groups at the end of the experiment were 7.5% lighter and 14.8% heavier, respectively, than that in the 23 °C group. This may be due to an increase in energy expenditure with the decrease in temperature (Chi & Wang, 2011; Hammond & Wunder, 1995). Moreover, voles also mobilized energy reserves, including fat free carcass and body fat mass, to meet the elevated energy requirements with the decrease in temperature. In general, an increase in expensive metabolic organ mass (i.e., liver, heart, small intestine, and kidneys) and small intestine length during cold acclimation are adaptive responses to elevated energy needs and food processing capability (Daan et al., 1990; Hammond & Wunder, 1995; Konarzewski & Diamond, 1995). In our study, the liver, heart, and kidney mass and small intestine length also increased significantly with the decline in temperature, consistent with previous studies (Chi & Wang, 2011; Zhang & Wang, 2006). These changes in metabolic organs would satisfy the increased energy requirements and food processing capability under cold exposure.

### Oxidative stress and gross energy intake

Energy requirements usually increase across many species in cold environments (Chi & Wang, 2011; Liu et al., 2009; Zhou et al., 2015). Thus, animals adopt different adaptive strategies, such as increased food and gross energy intake, as observed in the voles in our study. The increase in GEI with the decline in temperature also indicated an elevation in the metabolic rate of voles, although resting metabolic rate was not examined in our study. Previous research has shown that cold exposure significantly increases metabolic rate in voles (Li et al., 2001; Liu et al., 2009; Zhang & Wang, 2006). According to the rate of living-free radical hypothesis, higher metabolic rates, which are achieved by enhancing mitochondrial oxidative phosphorylation, should increase the production of free radicals (i.e., ROS) (Harman, 1956; Pearl, 1928; Selman et al., 2013; Speakman et al., 2004). In the current study, the increase in ROS production with the decrease in temperature, as indicated by H_2_O_2_ levels in the liver, was consistent with this hypothesis, whereas the reduction of ROS in the heart and unchanged ROS in the kidney, testis, and small intestine were not compatible with this hypothesis. The reason of these disparate results might lie in the fact that an elevated metabolic rate during cold acclimation does not necessarily result in greater ROS production (Costantini, 2008; Hulbert et al., 2007). In light of the “uncoupling to survive” hypothesis, increased uncoupling proteins in response to cold temperature can decrease ROS production by lowering the potential of the inner mitochondrial membrane (Brand, 2000; Speakman et al., 2004). Several studies have also demonstrated that an increased metabolic rate is not associated with elevated ROS levels in skeletal muscle or certain organs, including the liver, heart, lungs, spleen, kidneys, and digestive tract (Chen et al., 2014; Selman et al., 2002; Stier et al., 2014). The increased UCP1 content in voles upon cold exposure in previous studies might account for the ROS results in our experiment (Liu et al., 2009; Zhang & Wang, 2006).

In the present study, high temperature led to an increase in oxidation, as indicated by the H_2_O_2_ levels in the vole heart, which is consistent with previous research showing increased levels of oxidation at high temperature in broiler chickens (Tan et al., 2010). In light of the oxidation handicap hypothesis, testosterone may elevate the levels of ROS (Alonso-Alvarez et al., 2007, 2008). The increased ROS levels in voles under high temperature may also be caused by higher testosterone concentration, although no significant correlation was detected between H_2_O_2_ and testosterone levels.

In general, excessive ROS production can lead to oxidative damage (Burton & Jauniaux, 2011; Costantini, 2008; Finkel & Holbrook, 2000; Hulbert et al., 2007). In the present study, however, MDA content in the liver, heart, kidney, testis, and small intestine, which did not respond to high or low temperature treatment, was not related to ROS (H_2_O_2_) levels. This result agrees with several studies in which MDA content in the liver and serum of female Mongolian gerbils (Yang et al., 2013) or in the liver, heart, muscle, and brain of striped hamsters (Zhou et al., 2015) was not affected by high or low temperature treatment. This may be because the voles were able to maintain or increase SOD, T-AOC, and CAT activity in the five tissues, even under high or low temperature environments, which removed excessive free radicals.

### Antioxidant defense and hormone profiles

In the present study, CAT activity in the testis increased upon cold exposure but did not change under high temperature. Conversely, testosterone levels increased under high temperature but did not change with cold exposure. Thus, the effects of high temperature on the voles were different from those of low temperature.

Secretion of stress hormones such as corticosterone usually increase in animals facing stressful conditions, including high or low ambient temperature (Bligh-Tynan et al., 1993; Kim et al., 2013; Sapolsky et al., 2000). In the current study, however, corticosterone concentration in voles was not affected by high or low temperature, which is inconsistent with other studies showing increased corticosterone levels under cold stress (Adels et al., 1986; Shu et al., 1993). This discrepancy may be due to the difference in cold exposure duration. Secretion of corticosterone usually increases upon acute cold exposure, whereas its levels can recover to baseline under chronic cold exposure (Bligh-Tynan et al., 1993).

Several studies have shown that exogenous corticosterone administration can increase oxidative stress and decrease SOD activity in rats (Dhanabalan et al., 2010) and birds (Alonso-Alvarez et al., 2007; Lin et al., 2004). Here, we found that corticosterone concentration did not respond to high or low temperature treatment, and it was not correlated with ROS and MDA levels or SOD, CAT, and T-AOC activities in the liver, heart, kidney, testis, and small intestine (except for a negative correlation with SOD activity in the testis). Thus, it appears that corticosterone could not fully explain the impact of hot and cold temperature on oxidative stress and antioxidant capacity in voles.

The oxidation handicap hypothesis holds that testosterone incurs oxidative costs (Alonso-Alvarez et al., 2007, 2008). Although the testosterone concentration was significantly affected by temperature in the tested voles, it was not correlated with H_2_O_2_ levels, MDA content, or SOD, CAT, and T-AOC activities in the five tested tissues (i.e., liver, heart, kidney, testis, and small intestine), with the exception of a positive correlation with SOD activity in the testis. This result is consistent with earlier research demonstrating that increased testosterone does not cause oxidative damage to DNA but enhances total antioxidant capacity in yellowthroat warblers (*Geothlypis trichas*) (Taff & Freeman-Gallant, 2014). Several authors have argued that detection of oxidative costs is both tissue and assay-dependent (Yang et al., 2013), suggesting that additional parameters and tissues may have identified certain direct oxidative costs of testosterone in the present study.

### Immunity, body fat, and hormone profiles

Innate immunity, as indicated by bacteria killing capacity, was lower in the 4 °C group than in the 23 °C group. This may be due to the reduction of body fat in voles upon cold exposure. Adipose tissues are not only considered as endocrine and immune organs (Ahima & Flier, 2000; Trayhurn, 2005), but also as energy depots for expensive physiological processes, including immune function (Demas et al., 1997; Demas, 2004; Moret & Schmid-Hempel, 2000). Previous research has demonstrated that animals with low energy reserves choose to allocate less energy to immune defense than animals with higher reserves (Houston et al., 2007). Therefore, reductions in body fat can impair immunity (Chandra, 1996; Demas et al., 2003). In the face of energy demanding conditions, including cold environments, trade-offs may occur among different physiological processes. Voles might divert energy from less critical physiological functions such as immunity to more important processes such as thermogenesis for immediate survival during cold exposure (Liu et al., 2009; Zhang & Wang, 2006). However, further research is required to clarify whether trade-offs occur between thermogenic capacity and immune function under cold environments.

Both corticosterone and testosterone have suppressive effects on immunity (Marketon & Glaser, 2008; Sapolsky et al., 2000; Trigunaite et al., 2015). In the present study, however, we found no correlation between corticosterone and testosterone and innate immunity. Thus, these two hormones could not account for the variation in immunity observed in voles. Recently, several authors have shown that oxidative stress may also impair immune function (Costantini & Møller, 2009; Sordillo & Aitken, 2009). We found that innate immunity was negatively correlated with H_2_O_2_ levels in the liver and MDA content in the kidney. Suppression of innate immunity in the 4 °C group compared to the control may be caused by oxidative stress in voles.

In the current study, voles exhibited several adaptive strategies to cope with high and low temperature. Voles increased gross energy intake and active metabolic organs (i.e., heart, liver, kidneys) and mobilized energy reserves with the decline in temperature. However, voles may maintain or increase antioxidant defense at the cost of innate immunity under cold temperature. Therefore, up-regulation or maintenance of antioxidant defenses may be crucial for voles to survive under dramatically fluctuating ambient temperatures from the perspective of oxidative ecology (Boonstra, 2013).

## References

[ZoolRes-40-4-305-R1] Adels LE, Leon M, Wiener SG, Smith MS 1986 Endocrine response to acute cold exposure by lactating and non-lactating Norway rats. Physiology & Behavior, 36(1): 179–181. 3952179 10.1016/0031-9384(86)90093-4

[ZoolRes-40-4-305-R2] Ahima RS, Flier JS 2000 Adipose tissue as an endocrine organ. Trends in Endocrinology & Metabolism, 11(8): 327–332. 10996528 10.1016/s1043-2760(00)00301-5

[ZoolRes-40-4-305-R3] Alonso-Alvarez C, Bertrand S, Faivre B, Chastel O, Sorci G 2007 Testosterone and oxidative stress: the oxidation handicap hypothesis. Proceedings of the Royal Society B, 274(1161): 819–825. 17251089 10.1098/rspb.2006.3764 PMC2093982

[ZoolRes-40-4-305-R4] Alonso-Alvarez C, Perez-Rodriguez L, Mateo R, Chastel O, Vinuela J 2008 The oxidation handicap hypothesis and the carotenoid allocation trade-off. Journal of Evolutionary Biology, 21(6): 1789–1797. 18713241 10.1111/j.1420-9101.2008.01591.x

[ZoolRes-40-4-305-R5] Bligh-Tynan ME, Bhagwat SA, Castonguay TW 1993 The effects of chronic cold exposure on diurnal corticosterone and aldosterone rhythms in Sprague-Dawley rats. Physiology & Behavior, 54(2): 363–367. 8372133 10.1016/0031-9384(93)90124-x

[ZoolRes-40-4-305-R6] Boonstra R 2013 The ecology of stress: a marriage of disciplines. Functional Ecology, 27(1): 7–10.

[ZoolRes-40-4-305-R7] Brand MD 2000 Uncoupling to survive? The role of mitochondrial inefficiency in ageing. Experimental Gerontology, 35(6–7): 811–820. 11053672 10.1016/s0531-5565(00)00135-2

[ZoolRes-40-4-305-R8] Burton GJ, Jauniaux E 2011 Oxidative stress. Best Practice & Research Clinical Obstetrics & Gynaecology, 25(3): 287–299. 21130690 10.1016/j.bpobgyn.2010.10.016 PMC3101336

[ZoolRes-40-4-305-R9] Calder PC, Kew S 2002 The immune system: a target for functional foods? British Journal of Nutrition, 88: S165–176. 12495459 10.1079/BJN2002682

[ZoolRes-40-4-305-R10] Carroll JA, Burdick NC, Chase CC, Coleman SW, Spiers DE 2012 Influence of environmental temperature on the physiological, endocrine, and immune responses in livestock exposed to a provocative immune challenge. Domestic Animal Endocrinology, 43(2): 146–153. 22425434 10.1016/j.domaniend.2011.12.008

[ZoolRes-40-4-305-R11] Chandra RK 1996 Nutrition, immunity and infection: from basic knowledge of dietary manipulation of immune responses to practical application of ameliorating suffering and improving survival. Proceeding of the National Academy of Sciences of the United States of America, 93(25): 14304–14307. 10.1073/pnas.93.25.14304 PMC34479 8962043

[ZoolRes-40-4-305-R12] Chen ZZ 1998 Land form and climate in Xilin river valley. In: The Study of Grassland Ecosystem.Beijing, China: Science Press, 13–22.

[ZoolRes-40-4-305-R13] Chen KX, Wang CM, Wang GY, Zhao ZJ 2014 Energy budget, oxidative stress and antioxidant in striped hamster acclimated to moderate cold and warm temperatures. Journal of Thermal Biology, 44: 35–40. 25086971 10.1016/j.jtherbio.2014.06.005

[ZoolRes-40-4-305-R14] Chi QS, Wang DH 2011 Thermal physiology and energetics in male desert hamsters (*Phodopus roborovskii*) during cold acclimation. Journal of Comparative Physiology B, 181(1): 91–103. 10.1007/s00360-010-0506-6 20714728

[ZoolRes-40-4-305-R15] Costantini D 2008 Oxidative stress in ecology and evolution: lessons from avian studies. Ecology Letters, 11(11): 1238–1251. 18803642 10.1111/j.1461-0248.2008.01246.x

[ZoolRes-40-4-305-R16] Costantini D, Møller AP 2009 Does immune response cause oxidative stress in birds? A meta-analysis. Comparative Biochemistry and Physiology Part A, 153(3): 339–344. 10.1016/j.cbpa.2009.03.010 19303455

[ZoolRes-40-4-305-R17] Daan S, Masman D, Groenewold A 1990 Avian basal metabolic rates: their association with body composition and energy expenditure in nature. American Journal of Physiology-Regulatory, Integrative and Comparative Physiology, 259(2): R333–340. 10.1152/ajpregu.1990.259.2.R333 2386245

[ZoolRes-40-4-305-R18] Del Rio D, Stewart AJ, Pellegrini N 2005 A review of recent studies on malondialdehyde as toxic molecule and biological marker of oxidative stress. Nutrition, Metabolism and Cardiovascular Diseases, 15(4): 316–328. 10.1016/j.numecd.2005.05.003 16054557

[ZoolRes-40-4-305-R19] Demas GE 2004 The energetics of immunity: a neuroendocrine link between energy balance and immune function. Hormones and Behavior, 45(3): 173–180. 15047012 10.1016/j.yhbeh.2003.11.002

[ZoolRes-40-4-305-R20] Demas GE, Drazen DL, Nelson RJ 2003 Reductions in total body fat decrease humoral immunity. Proceedings of the Royal Society B, 270(1518): 905–911. 12803904 10.1098/rspb.2003.2341 PMC1691330

[ZoolRes-40-4-305-R21] Demas GE, Chefer V, Talan MI, Nelson RJ 1997 Metabolic costs of mounting an antigen-stimulated immune response in adult and aged C57BL/6J mice. American Journal of Physiology–Regulatory, Integrative and Comparative Physiology, 273(5): 1631–1637. 10.1152/ajpregu.1997.273.5.R1631 9374803

[ZoolRes-40-4-305-R22] Demas GE, Zysling DA, Beechler BR, Muehlenbein MP, French SS 2011 Beyond phytohaemagglutinin: assessing vertebrate immune function across ecological contexts. Journal of Animal Ecology, 80(4): 710–730. 21401591 10.1111/j.1365-2656.2011.01813.x

[ZoolRes-40-4-305-R23] Dhanabalan S, Dćruz SC, Mathur PP 2010 Effects of corticosterone and 2, 3, 7, 8-tetrachloro-dibenzo-p-dioxin on epididymal antioxidant system in adult rats. Journal of Biochemical and Molecular Toxicology, 24(4): 242–249. 20806395 10.1002/jbt.20332

[ZoolRes-40-4-305-R24] Dickinson BC, Chang CJ 2011 Chemistry and biology of reactive oxygen species in signaling or stress responses. Nature Chemical Biology, 7(8): 504–511. 21769097 10.1038/nchembio.607 PMC3390228

[ZoolRes-40-4-305-R25] Dowling DK, Simmons LW 2009 Reactive oxygen species as universal constraints in life-history evolution. Proceedings of the Royal Society B, 276(1663): 1737–1745. 19324792 10.1098/rspb.2008.1791 PMC2674489

[ZoolRes-40-4-305-R26] Fantuzzi G 2005 Adipose tissue, adipokines, and inflammation. Journal of Allergy and Clinical Immunology, 115(5): 911–919. 15867843 10.1016/j.jaci.2005.02.023

[ZoolRes-40-4-305-R27] Finkel T, Holbrook NJ 2000 Oxidants, oxidative stress and the biology of ageing. Nature, 408: 239–247. 11089981 10.1038/35041687

[ZoolRes-40-4-305-R28] Grodzinski W, Wunder BA 1975 Ecological energetics of small mammals. In: Golley FB, Petrusewicz K, Ryszkowski L (eds). Small Mammals: Their Productivity and Population Dynamics. Cambridge: Cambridge University Press, 173–204.

[ZoolRes-40-4-305-R29] Hall ME, Blount JD, Forbes S, Royle NJ 2010 Does oxidative stress mediate the trade-off between growth and self-maintenance in structured families? *Functional Ecology* , 24(2): 365–373.

[ZoolRes-40-4-305-R30] Hammond KA, Wunder BA 1995 Effect of cold temperatures on the morphology of gastrointestinal tracts of two microtine rodents. Journal of Mammalogy, 76(1): 232–239.

[ZoolRes-40-4-305-R31] Harman D 1956 Aging: a theory based on free radical and radiation chemistry. Journal of Gerontology, 11(3): 298–300. 13332224 10.1093/geronj/11.3.298

[ZoolRes-40-4-305-R32] Houston AI, McNamara JM, Barta Z, Klasing KC 2007 The effect of energy reserves and food availability on optimal immune defence. Proceedings of the Royal Society B, 274(1627): 2835–2842. 17848371 10.1098/rspb.2007.0934 PMC2373797

[ZoolRes-40-4-305-R33] Hulbert AJ, Pamplona R, Buffenstein R, Buttemer WA 2007 Life and death: metabolic rate, membrane composition, and life span of animals. Physiological Reviews, 87(4): 1175–1213. 17928583 10.1152/physrev.00047.2006

[ZoolRes-40-4-305-R34] Kanter M, Aktas C, Erboga M 2013 Heat stress decreases testicular germ cell proliferation and increases apoptosis in short term: an immunohistochemical and ultrastructural study. Toxicology and Industrial Health, 29(2): 99–113. 22082826 10.1177/0748233711425082

[ZoolRes-40-4-305-R35] Kim HG, Lee JS, Han JM, Lee JS, Choi MK, Son SW, Son CG 2013 Myelophil attenuates brain oxidative damage by modulating the hypothalamus-pituitary-adrenal (HPA) axis in a chronic cold-stress mouse model. Journal of Ethnopharmacology, 148(2): 505–514. 23665312 10.1016/j.jep.2013.04.046

[ZoolRes-40-4-305-R36] King DA 2004 Climate change science: adapt, mitigate, or ignore?. Science, 303(5655): 176–177. 14715997 10.1126/science.1094329

[ZoolRes-40-4-305-R37] Konarzewski M, Diamond J 1995 Evolution of basal metabolic rate and organ masses in laboratory mice. Evolution, 49(6): 1239–1248. 28568534 10.1111/j.1558-5646.1995.tb04450.x

[ZoolRes-40-4-305-R38] Kozyreva TV, Eliseeva LS 2000 Immune response in cold exposures of different types. Journal of Thermal Biology, 25(5): 401–404. 10838180 10.1016/s0306-4565(99)00113-8

[ZoolRes-40-4-305-R39] Kozyreva TV, Eliseeva LS 2004 The immune system response to antigen in cold- and warm-adapted rats. Journal of Thermal Biology, 29(7–8): 865–869.

[ZoolRes-40-4-305-R40] Li YG, Yan ZC, Wang DH 2010 Physiological and biochemical basis of basal metabolic rates in Brandt’s voles (*Lasiopodomys brandtii*) and Mongolian gerbils (*Meriones unguiculatus*). Comparative Biochemistry and Physiology Part A, 157(3): 204–211. 10.1016/j.cbpa.2010.06.183 20601053

[ZoolRes-40-4-305-R41] Li QF, Sun RY, Huang CX, Wang ZK, Liu XT, Hou JJ, Liu JS, Cai LQ, Li N, Zhang SZ, Wang Y 2001 Cold adaptive thermogenesis in small mammals from different geographical zones of China. Comparative Biochemistry and Physiology Part A, 129(4): 949–961. 10.1016/s1095-6433(01)00357-9 11440879

[ZoolRes-40-4-305-R42] Lin H, Decuypere E, Buyse J 2004 Oxidative stress induced by corticosterone administration in broiler chickens (*Gallus gallus domesticus*). Comparative Biochemistry and Physiology Part B, 139(4): 745–751. 10.1016/j.cbpc.2004.09.014 15581807

[ZoolRes-40-4-305-R43] Liu H, Wang DH, Wang ZW 2003 Energy requirements during reproduction in female Brandt’s voles (*Microtus brandtii*). Journal of Mammalogy, 84(4): 1410–1416.

[ZoolRes-40-4-305-R44] Liu JS, Yang M, Sun RY, Wang DH 2009 Adaptive thermogenesis in Brandt’s vole (*Lasiopodomys brandtii*) during cold and warm acclimation. Journal of Thermal Biology, 34(2): 60–69.

[ZoolRes-40-4-305-R45] Lomakina LV 1980 Effects of low temperature on lipid peroxidation and intensity of proteolysis in rat brain and liver. Ukrainskii Biokhimicheskii Zhurnal, 52(3): 305–308. 6992368

[ZoolRes-40-4-305-R46] Marketon JIW, Glaser R 2008 Stress hormones and immune function. Cellular Immunology, 252(1–2): 16–26. 18279846 10.1016/j.cellimm.2007.09.006

[ZoolRes-40-4-305-R47] Marnila P, Lilius E 2015 Thermal acclimation in the perch (*Perca fluviatilis L* .) immunity. Journal of Thermal Biology, 54: 47–55. 26615726 10.1016/j.jtherbio.2015.01.002

[ZoolRes-40-4-305-R48] Marri V, Richner H 2015 Immune response, oxidative stress and dietary antioxidants in great tit nestlings. Comparative Biochemistry and Physiology Part A, 179: 192–196. 10.1016/j.cbpa.2014.10.013 25446145

[ZoolRes-40-4-305-R49] Martin LB, Scheuerlein A, Wikelski M 2003 Immune activity elevates energy expenditure of house sparrows: a link between direct and indirect costs? Proceedings of the Royal Society B, 270(1511): 153–158. 12590753 10.1098/rspb.2002.2185 PMC1691219

[ZoolRes-40-4-305-R50] Martin LB, Weil ZM, Nelson RJ 2007 Seasonal changes in vertebrate immune activity: mediation by physiological trade-offs. Philosophical Transactions of the Royal Society B, 363(1490): 1–19. 10.1098/rstb.2007.2142 PMC2606753 17638690

[ZoolRes-40-4-305-R51] Metcalfe NB, Alonso-Alvarez C 2010 Oxidative stress as a life-history constraint: the role of reactive oxygen species (ROS) in shaping phenotypes from conception to death. Functional Ecology, 24(5): 984–996.

[ZoolRes-40-4-305-R52] Monaghan P, Metcalfe NB, Torres R 2009 Oxidative stress as a mediator of life history trade-offs: mechanisms, measurements and interpretation. Ecology Letters, 12(1): 75–92. 19016828 10.1111/j.1461-0248.2008.01258.x

[ZoolRes-40-4-305-R53] Moret Y, Schmid-Hempel P 2000 Survival for immunity: the price of immune system activation for bumblebee workers. Science, 290(5494): 1166–1168. 11073456 10.1126/science.290.5494.1166

[ZoolRes-40-4-305-R54] Owens IPF, Wilson K 1999 Immunocompetence: a neglected life history trait or conspicuous red herring? Trends in Ecology & Evolution, 14(5): 170–172.

[ZoolRes-40-4-305-R55] Pearl R 1928 The Rate of Living. London, UK: University of London Press.

[ZoolRes-40-4-305-R56] Pei YX, Wang DH, Hume ID 2001 Effects of dietary fibre on digesta passage, nutrient digestibility, and gastrointestinal tract morphology in the granivorous Mongolian gerbil (*Meriones unguiculatus*). Physiological and Biochemical Zoology, 74(5): 742–749. 11517459 10.1086/322928

[ZoolRes-40-4-305-R57] Raut AM, Suryakar AN, Mhaisekar D 2012 Study of oxidative stress in relation with antioxidant status in chronic bronchitis. International Journal of Public Health Science, 1(1): 7–10.

[ZoolRes-40-4-305-R58] Sapolsky RM, Romero LM, Munck AU 2000 How do glucocorticoids influence stress responses? Integrating permissive, suppressive, stimulatory, and preparative actions. Endocrine Reviews, 21(1): 55–89. 10696570 10.1210/edrv.21.1.0389

[ZoolRes-40-4-305-R59] Selman C, McLaren JS, Himanka MJ, Speakman JR 2000 Effect of long-term cold exposure on antioxidant enzyme activities in a small mammal. Free Radical Biology and Medicine, 28(8): 1279–1285. 10889458 10.1016/s0891-5849(00)00263-x

[ZoolRes-40-4-305-R60] Selman C, McLaren JS, Collins AR, Duthie GG, Speakman JR 2008 The impact of experimentally elevated energy expenditure on oxidative stress and lifespan in the short-tailed field vole *Microtus agrestis* . Proceedings of the Royal Society B, 275(1645): 1907–1916. 18467297 10.1098/rspb.2008.0355 PMC2596373

[ZoolRes-40-4-305-R61] Selman C, McLaren JS, Collins AR, Duthie GG, Speakman JR 2013 Deleterious consequences of antioxidant supplementation on lifespan in a wild-derived mammal. Biology Letters, 9(4): 1–4. 10.1098/rsbl.2013.0432 PMC3730656 23825087

[ZoolRes-40-4-305-R62] Selman C, Grune T, Stolzing A, Jakstadt M, Mclaren JS, Speakman JR 2002 The consequences of acute cold exposure on protein oxidation and proteasome activity in short-tailed field voles, *Microtus agrestis* . Free Radical Biology and Medicine, 33(2): 259–265. 12106821 10.1016/s0891-5849(02)00874-2

[ZoolRes-40-4-305-R63] Sheldon BC, Verhulst S 1996 Ecological immunology: costly parasite defences and trade-offs in evolutionary ecology. Trends Ecology & Evolution, 11(8): 317–321. 21237861 10.1016/0169-5347(96)10039-2

[ZoolRes-40-4-305-R64] Shu J, Stevenson JR, Zhou X 1993 Modulation of cellular immune responses by cold water swim stress in the rat. Developmental & Comparative Immunology, 17(4): 357–371. 8375569 10.1016/0145-305x(93)90007-d

[ZoolRes-40-4-305-R65] Smith KG, Hunt JL 2004 On the use of spleen mass as a measure of avian immune system strength. Oecologia, 138(1): 28–31. 14576931 10.1007/s00442-003-1409-y

[ZoolRes-40-4-305-R66] Sordillo LM, Aitken SL 2009 Impact of oxidative stress on the health and immune function of dairy cattle. Veterinary Immunology and Immunopathology, 128(1–3): 104–109. 19027173 10.1016/j.vetimm.2008.10.305

[ZoolRes-40-4-305-R67] Speakman JR, Talbot DA, Selman C, Snart S, McLaren JS, Redman P, Krol E, Jackson DM, Johnson MS, Brand MD 2004 Uncoupled and surviving: individual mice with high metabolism have greater mitochondrial uncoupling and live longer. Aging Cell, 3(3): 87–95. 15153176 10.1111/j.1474-9728.2004.00097.x

[ZoolRes-40-4-305-R68] Stier A, Bize P, Habold C, Bouillaud F, Massemin S, Criscuolo F 2014 Mitochondrial uncoupling prevents cold-induced oxidative stress: a case study using UCP1 knockout mice. Journal of Experimental Biology, 217: 624–630. 24265420 10.1242/jeb.092700

[ZoolRes-40-4-305-R69] Taff CC, Freeman-Gallant CR 2014 An experimental test of the testosterone mediated oxidation handicap hypothesis in a wild bird. Hormones and Behavior, 66(2): 276–282. 24907452 10.1016/j.yhbeh.2014.05.006

[ZoolRes-40-4-305-R70] Tan GY, Yang L, Fu YQ, Feng JH, Zhang MH 2010 Effects of different acute high ambient temperatures on function of hepatic mitochondrial respiration, antioxidative enzymes, and oxidative injury in broiler chickens. Poultry Science, 89(1): 115–122. 10.3382/ps.2009-00318 20008809

[ZoolRes-40-4-305-R71] Tieleman BI, Williams JB, Ricklefs RE, Klasing KC 2005 Constitutive innate immunity is a component of the pace-of-life syndrome in tropical birds. Proceedings of the Royal Society B, 272(1573): 1715–1720. 16087427 10.1098/rspb.2005.3155 PMC1559858

[ZoolRes-40-4-305-R72] Trayhurn P 2005 Endocrine and signalling role of adipose tissue: new perspectives on fat. Acta Physiologica, 184(4): 285–293. 10.1111/j.1365-201X.2005.01468.x 16026420

[ZoolRes-40-4-305-R73] Trigunaite A, Dimo J, Jørgensen TN 2015 Suppressive effects of androgens on the immune system. Cellular Immunology, 294(2): 87–94. 25708485 10.1016/j.cellimm.2015.02.004

[ZoolRes-40-4-305-R74] Vasilijević A, Buzadžić B, Korać A, Petrović V, Janković A, Mićunović K, Korać B 2007 The effects of cold acclimation and nitric oxide on antioxidative enzymes in rat pancreas. Comparative Biochemistry and Physiology Part C, 145(4): 641–647. 17395542 10.1016/j.cbpc.2007.02.013

[ZoolRes-40-4-305-R75] Walker EP 1968 Mammals of the World.

[ZoolRes-40-4-305-R76] Baltimore: Johns Hopkins Press.

[ZoolRes-40-4-305-R77] Wang DH 2007 Advances in Animal Ecology. 1st ed. Beijing: Higher Education Press, 29–46.

[ZoolRes-40-4-305-R78] Wang DH, Wang YS, Wang ZW 2000 Metabolism and thermoregulation in the Mongolian gerbil *Meriones unguiculatus* . Acta Theriologica, 45(2): 183–192.

[ZoolRes-40-4-305-R79] Wang DH, Wang ZW, Wang YS, Yang JC 2003 Seasonal changes of thermogenesis in Mongolian gerbils (*Meriones unguiculatus*) and Brandt’s voles (*Microtus brandtii*). Comparative Biochemistry and Physiology Part A, 134: S96.

[ZoolRes-40-4-305-R80] Xu DL, Wang DH 2010 Fasting suppresses T cell-mediated immunity in female Mongolian gerbils (*Meriones unguiculatus*). Comparative Biochemistry and Physiology Part A, 155(1): 25–33. 10.1016/j.cbpa.2009.09.003 19748595

[ZoolRes-40-4-305-R81] Xu DL, Wang DH 2011 Glucose supplement reverses the fasting-induced suppression of cellular immunity in Mongolian gerbils (*Meriones unguiculatus*). Zoology, 114(5): 306–312. 21885265 10.1016/j.zool.2011.04.002

[ZoolRes-40-4-305-R82] Xu Y, Yang Z, Su C 1992 Enhancement of cellular immune function during cold adaptation of BALBc inbred mice. Cryobiology, 29(3): 422–427. 1499325 10.1016/0011-2240(92)90044-3

[ZoolRes-40-4-305-R83] Xu DL, Liu XY, Wang DH 2011 Food restriction and refeeding have no effect on cellular and humoral immunity in Mongolian gerbils (*Meriones unguiculatus*). Physiological and Biochemical Zoology, 84(1): 87–98. 21142689 10.1086/657687

[ZoolRes-40-4-305-R84] Yang XP 2004 Laboratory Manual in Animal Physiology. 1st ed. Beijing: Higher Education Press, 91–94.

[ZoolRes-40-4-305-R85] Yang DB, Xu YC, Wang DH, Speakman JR 2013 Effects of reproduction on immuno-suppression and oxidative damage, and hence support or otherwise for their roles as mechanisms underpinning life history trade-offs, are tissue and assay dependent. Journal of Experimental Biology, 216: 4242–4250. 23997195 10.1242/jeb.092049

[ZoolRes-40-4-305-R86] Yuksel S, Dilek A, Ozfer Y 2008 Antioxidative and metabolic responses to extended cold exposure in rats. Acta Biologica Hungarica, 59(1): 57–66. 18401945 10.1556/ABiol.59.2008.1.5

[ZoolRes-40-4-305-R87] Zhang XY, Wang DH 2006 Energy metabolism, thermogenesis and body mass regulation in Brandt’s voles (*Lasiopodomys brandtii*) during cold acclimation and rewarming. Hormones and Behavior, 50(1): 61–69. 16515788 10.1016/j.yhbeh.2006.01.005

[ZoolRes-40-4-305-R88] Zhang ZB, Wang ZW 1998 Ecology and Management of Rodent Pests in Agriculture. Beijing: Ocean Publishing House.

[ZoolRes-40-4-305-R89] Zhao ZJ, Wang DH 2006 Short photoperiod influences energy intake and serum leptin level in Brandt's voles (*Microtus brandtii*). Hormones and Behavior, 49(4): 463–469. 16293255 10.1016/j.yhbeh.2005.10.003

[ZoolRes-40-4-305-R90] Zhou SS, Cao LL, Xu WD, Cao J, Zhao ZJ 2015 Effect of temperature on oxidative stress, antioxidant levels and uncoupling protein expression in striped hamsters. Comparative Biochemistry and Physiology Part A, 189: 84–90. 10.1016/j.cbpa.2015.07.017 26244518

